# Revisiting Mental Simulation in Language Comprehension: Six Replication Attempts

**DOI:** 10.1371/journal.pone.0051382

**Published:** 2012-12-26

**Authors:** Rolf A. Zwaan, Diane Pecher

**Affiliations:** Psychology Department, Erasmus University Rotterdam, Rotterdam, The Netherlands; National Taiwan University, Taiwan

## Abstract

The notion of language comprehension as mental simulation has become popular in cognitive science. We revisit some of the original empirical evidence for this. Specifically, we attempted to replicate the findings from earlier studies that examined the mental simulation of object orientation, shape, and color, respectively, in sentence-picture verification. For each of these sets of findings, we conducted two web-based replication attempts using Amazon's Mechanical Turk. Our results are mixed. Participants responded faster to pictures that matched the orientation or shape implied by the sentence, replicating the original findings. The effect was larger and stronger for shape than orientation. Participants also responded faster to pictures that matched the color implied by the sentence, whereas the original studies obtained *mis*match advantages. We argue that these results support mental simulation theory, show the importance of replication studies, and show the viability of web-based data collection.

## Introduction

The past decade has seen a shift in how language comprehension, and, in fact, all of cognition is conceptualized. The working assumption up to about 15 years ago had been that the human mind manipulates abstract, arbitrary, and amodal symbols and that this combination of manipulation and symbolic representations constituted cognition. However, this kind of view suffers from the grounding problem [1]. Abstract, amodal, and arbitrary symbols have no connection to actual experience; they are floating free in some mental ether and are therefore essentially meaningless. If we want to develop serious theories of cognition, Harnad argued, these theories need symbols that are grounded in perception and action. This idea was echoed in several later papers [2],[3]. Focusing on language, Barsalou argued that language comprehension should not be viewed as the disembodied manipulation of symbols, the way a computer might do it. Rather, cognition should be viewed as the mental simulation of events by reactivating traces of earlier experiences. A large number of studies in psychology and neuroscience have addressed these issues over the past 12 years.

Although the notion of mental simulation as proposed by Barsalou was appealing to some researchers at the time, including the authors of this article, it was very much a theory in search of evidence. In his article, Barsalou provides a “Gedankenexperiment,” a thought experiment. In a mental simulation, he proposed, there should be a difference between “the pencil is in the cup” and “the pencil is in the drawer.” In the first case, the pencil is in a vertical position but in the second sentence it is horizontal. Barsalou argued that the pencil's orientation should be an automatic by-product of a mental simulation, whereas it is not part of an abstract, amodal, and arbitrary mental representation. For example, the propositional representation [IN[PENCIL,CUP]] does not contain information about the pencil's orientation and according to amodal theories such elaborative inferences would not be made or they would require cumbersome logical formulae.

When Zwaan and his students discussed this article during a lab meeting, one of his graduate students, Rob Stanfield, came up with a way to test this idea: by using a sentence-picture verification task. The idea is simple but elegant. Participants read a sentence in which the orientation of an object is implied, rather than stated explicitly, and then they decide whether the object shown in the subsequently presented picture was mentioned in the sentence. The key feature of the paradigm is that the orientation of the pictured object is manipulated; it is either horizontal or vertical. This means that the pictured object's orientation either matches or mismatches the orientation of the object as it was implied by the sentence. If language comprehenders perform mental simulations, they should show sensitivity to this difference in orientation. The results were as predicted by Barsalou's simulation account. Participants were faster to verify pictures that matched the implied orientation than pictures that mismatched [4].

Crucial to the task is that the pictured object's orientation is irrelevant. The participants merely indicate if the object was mentioned in the sentence or not, a very simple task given that it is blatantly obvious when the pictured object is not mentioned in the sentence. With the task set up like this, participants might be able to perform well without understanding the sentence. They merely have to keep track of the nouns in the sentence without actually attempting to comprehend the sentences. To ensure that participants engage in some minimal form of comprehension, they are prompted at irregular intervals to recall a previously seen sentence or answer a question about it. Importantly, to prevent tipping the participants off about the purpose of the experiment, these tasks do not require the participants to think about the orientation of the target object.

If comprehenders perform mental simulations, then other visual dimensions should also be simulated. A subsequent series of experiments [5] examined whether the match effect extends to visual shape, arguably a more salient visual dimension than orientation (e.g., [6]). They used the sentence-picture verification paradigm (in Experiment 1) and sentences such as “The eagle was in the sky” and “The eagle was in the nest,” in which the shape of the target entity was implied. These sentences were followed by a line drawing of either an eagle with its wings stretched out or one with its wings drawn in. As in the orientation study, a significant match effect was obtained, which appeared more robust than the orientation effect, as indicated by the effect sizes in Table 1.

**Table 1:** Overview of Match Effects (Median RTs) in Experiments 1a to 3b and in Previous Studies.

	N	MatchM (SD)	MismatchM (SD)	Difference	*p*	Effect size (Cohen's *d*)	*BF_01_*
**Orientation**							
Experiment 1a	164	931 (318)	964 (354)	33	.020	.10	1.06
Experiment 1b	172	982 (382)	1020 (422)	38	.010	.09	0.46
Stanfield & Zwaan (2001)	40	838 (331)	882 (329)	44	.016	.13	
1a and 1b combined							0.04
**Shape**							
Experiment 2a	176	979 (356)	1036 (404)	57	.0002	.15	0.02
Experiment 2b	176	1056 (361)	1126 (404)	70	.0001	.18	0.01
Zwaan et al. (2002, Exp. 1)	42	697 (202)	761 (210)	64	.0008	.31	
2a and 2b combined							0.01
**Color**							
Experiment 3a	152	1221 (549)	1378 (750)	157	.0001	.24	0.01
Experiment 3b	152	1207 (395)	1292 (577)	85	.0237	.17	1.22
Connell (2007)	42	1369 (638)	1215 (509)	−154	.0039	−.27	
Connell (2005)	60	1328 (577)	1190 (542)	−138	.0080	−.25	
3a and 3b combined							.01

Other researchers have used the sentence-picture verification paradigm to study other visual dimensions. One such dimension is color. Connell [7],[8] presented participants with sentences such as “John looked at the steak in the butcher's window” followed by a picture of a red (match) or brown (mismatch) steak. Surprisingly, in light of the earlier findings, Connell obtained significantly faster responses to the *mismatching* than to the matching items, which she nevertheless interpreted as support for mental simulation theory. Connell [8] argued that color, as opposed to orientation and shape, is an unstable object property (for example, it can only be perceived unimodally), which leads to a different pattern of activation (for a detailed explanation, see [8]).

Our goal in this article is to take a step back and revisit these experiments by performing exact replications. This goal is motivated by three developments. First, we agree with an increasing number of voices in the literature (e.g., [9],[10],[11],[12]) that express concern about the lack of replications in the field. The field of psychology in general—and perhaps the area of embodied cognition in particular—is aimed at producing novel findings. This is understandable. For example, in the case of embodied cognition, there was virtually no research 15 years ago, so that people became naturally interested in exploring the generalizability of the early findings. If it is found that comprehenders mentally simulate the orientation of objects, then wouldn't they also simulate shape, color, motion, and perspective (to name just a few topics)? However, the downside of such a novelty-seeking approach is that the original findings, which may have spawned a large number of follow-up studies, are never exactly replicated. As a result, it is not clear whether the literature rests on a firm base. Our aim here is to examine part of the firmness of the empirical base for mental simulation in language comprehension.

Why did we select these three studies for replication, apart from the fact that they all focus on aspects of the same topic, mental simulations in language comprehension? What is their “replication value”? One criterion for assessing replication value is the impact that a particular study has on the field. All three studies were published in major journals. Both [4] and [5] have been cited quite often. As of October 15, 2012 they have been cited 135 and 160 times in the Web of Science and 324 and 368 times in Google Scholar, respectively. The color study [8] is younger and has not yet received a large number of citations. Its replication value derives in an important part from the fact that its results run counter to theoretical predictions and earlier findings.

One can think of exact and conceptual replications as being on a continuum, where by one endpoint (“exact”) can only be approximated. No experiment can use exactly the same participants in exactly the same mental states and environmental conditions as an earlier experiment [11]. However, the experiment can use the same stimuli, the same instructions, the same procedure and similar participants to approximate an exact replication. Conceptual replications have the appeal that they show the generalizability of an effect (and the added appeal making the associated paper easier to publish) but the disadvantage is that it is sometimes not clear whether the same phenomenon is tested across different studies. There also occurs a certain bias. If the study shows the effect in the expected direction, the conceptual replication is deemed successful. If not, then it is concluded that the phenomenon addressed in the second study is, after all, different from that in the first study. On the other hand, exact replications have the disadvantage that they might replicate an effect that is really due to a quirk in the stimulus materials, rather than a genuine empirical manifestation of the phenomenon under investigation. Thus, a combination of exact and conceptual replications is ideal. Given the current paucity of exact replications, however, we decided to try to replicate the original studies with the same materials.

The second reason motivating this article is that the state of affairs regarding mental simulations in language comprehension is a little puzzling. Why would a study of orientation show a modest effect, a study on shape a more robust effect, and a study on color the opposite effect? Our goal is to address these questions and suggest further avenues of research.

The third impetus for this research is more mundane. Occasionally people have approached us at conferences claiming some of these results are difficult to replicate, whereas others appear to be more easily replicable. Failures to replicate can have various reasons. First, failures to replicate are a statistical possibility, either because the original finding was a type I error or the replication was a type II error. Second, it could be that the replication attempt was performed inexpertly. Third, it could be that certain unknown factors contributed to finding an effect in the original study but not in a replication.

A look at the published literature shows that there are relatively few empirical studies investigating mental simulation of object orientation along the lines of [4]. A recent example is [13], who used a Dutch version of the orientation and shape stimuli in both a listening and reading comprehension experiment with Dutch 2^nd^ to 6^th^ graders. There are quite a few more mental simulation studies of shape, several of which were initiated by Zwaan and his colleagues. Their main reason for focusing on shape is that while the orientation manipulation seems more elegant, the first studies suggested that the shape effect is more robust, and therefore seemed a better tool to investigate the role of such factors as age [14], working memory span [15], negation [16], and comprehension skill [13] (although orientation was as strong a factor as was shape in this study) in mental simulation. Each of these studies showed significant match effects. Other research groups, investigating expertise [17], hemispheric differences [18], and visual spatial frequency [19] have also found the shape effect (limiting it to domain experts, the left hemisphere, and low spatial frequency, respectively). Recent unpublished data by Rommers, Meyer, and Huettig replicated the match effect for shape but not for orientation, although it should be noted that their study had low power to detect an effect and they had intermixed orientation and shape items, thus providing no exact replication. In short, the shape match advantage has been replicated a number of times but the evidence for the orientation match advantage is weaker.

It might be argued that having each sentence followed by a picture engenders unusual comprehension strategies on the part of the participants. However, three recent studies, each using a different method, showed that similar effects occurred when sentences were not followed by pictures immediately but were separated by considerable spans of time. More accurate recognition of match than mismatch pictures for both shape and orientation was found when there was a 45-minute delay between sentence reading and picture verification [20]. The match advantage was found even when pictures preceded sentences. Elevated eye-fixation times were found for sentences that mismatched the orientation of pictures presented 20 min earlier as part of an ostensibly unrelated experiment [21]. Finally, significant modulations of the N400 response in event-related potentials (ERP) were found when participants read sentences that mismatched the shape of pictured objects presented as part of an ostensibly unrelated experiment [22]. These findings indicate that the shape match effect is not due to strategies that might be invoked by alternating sentence reading and picture verification on every trial.

The color effect is younger than orientation and shape and has only been observed in two studies to our knowledge [7],[8]. Connell obtained faster picture verification responses when the color mismatched than when it matched. It is puzzling that the effect of color is the reverse of that of orientation and shape. Connell proposed that color is processed differently than other visual features such as shape. Color might be less important than shape for identifying objects [23]. As we see it, this might predict a smaller effect, but not a reversal of the match advantage. Moreover, several studies have suggested that color information is activated during object processing [24],[25],[26],[27] in a similar fashion as shape information [28]. There was a positive priming effect between names of objects that shared color, but only if color was made relevant by a prior task [29]. Priming of *shape*-related words, however, also depended on prior activation of shape information [30]. Thus, color might be less important than shape, but not to a large extent, and it is not clear that it is sufficiently different from shape and orientation so as to explain a negative match effect. To clarify these issues we investigated the robustness and direction of match effects for the three visual properties orientation, shape, and color.

An additional question is whether these effects are related to imagery or other individual differences. Although imagery ability and imagery preference are related to cognitive processes if they involve similar operations [31],[32],[33],[34], some studies have found no relation between imagery ability and effect size in paradigms that investigated mental simulations [4],[35]. Although it seems reasonable to assume that imagery and mental simulation share some processing, especially those involved in perception, imagery is based largely on conscious, effortful processes aimed at solving a difficult task. Mental simulation, on the other hand, might be a set of mostly unconscious processes that are recruited automatically for conceptual processing. Moreover, while individuals may differ in how much and what type of mental imagery they recruit, mental simulation during language comprehension might be a more universal mechanism and thus show less individual variation. On the other hand, the typical participant in psychology experiments might not be as representative of the general population as one would hope. Undergraduate students are a homogeneous group in terms of age and educational levels, and may even comprise an unusual group in some respects [36].

To sum up, we report a series of experiments in which we investigated the robustness of these three effects. We planned to replicate each experiment twice with a sample from MTurk that included more diverse ages and educational levels. In addition, we were interested in whether individual differences could be explained by imagery ability, age, or educational level. For this reason, we investigated the correlations between individual match-mismatch effect sizes and scores on imagery questionnaires, age, and education.

In the following experiments, we provide two identical replication attempts each for orientation (Experiments 1a and 1b) for which a significant match advantage was previously obtained [4], shape (Experiments 2a and 2b), for which a significant match advantage was previously obtained [4], and color (Experiments 3a and 3b), for which significant mismatch advantages were previously obtained [7],[8]. In each of the replication attempts, we used the same experimental stimuli and procedures as in the original experiments. Unfortunately, we did not have the exact filler items and comprehension questions of the original studies anymore. For each set, we created new filler items that matched the experimental sentences in structure and length and the experimental pictures in style and color. The comprehension questions were similar as those used by [4]. We made the comprehension questions comparable between experiments so that the difficulty level would be similar between experiments. In a departure from the original experiments, our replications were web based. This allowed us to use large numbers of participants and draw some conclusions about the generalization of research findings beyond the psychological laboratory. In Appendix S1 we discuss our use of web-based experiments in greater detail.

In each of the six experiments below, we used the same participant-recruitment and participant exclusion plan. We recruited 200 participants for each experiment. Of these, a large percentage completed the experiment. We were interested in running only native speakers of English. However, screening for native speakers in MTurk is counterproductive because nonnative speakers might falsely indicate that they are native speakers if they are interested in participating in the experiment. To prevent this problem, we asked participants about their native language at the end of the experiment as part of a demographic questionnaire. We excluded nonnative speakers of English; these comprised typically a small number, <10, in each experiment. We then excluded the small number of participants who either appeared to have reversed the response keys, having accuracy scores between .00 and .20. We then excluded participants who appeared not to have reversed the response keys but who had unusually low accuracy scores (<.80). As these exclusion procedures often left us with unequal number of participants per counterbalancing list, we eliminated the last-run participants of the longer list to create equal-length lists.

In all experiments, we analyzed the data using the same trimming procedures as those that were used in the original studies. [4] and [5] used the median RT per condition. [7],[8] trimmed the RTs by removing all RTs faster than 300 and slower than 3000 ms and then removed responses that were more than 2 standard deviations from the participant's mean in that condition. Because of problems associated with standard significance testing, especially when using large samples [37],[38],[39], we also calculated the posterior probability favoring the alternative hypothesis (i.e., the evidence for a match effect) using the JZS Bayes Factor(*BF_01_*, calculated with Rouder's web based application at http://pcl.missouri.edu/bayesfactor), which provides the odds ratio for the null/alternative hypotheses given the data (where 1 means that they are equally likely, larger values indicate more evidence for the null, and smaller values indicate more evidence for the alternative).

### Ethics Statement

The participants in all six experiments were recruited online and voluntarily subscribed for participation in all of the described experiments. Written consent was not obtained. This was waived by the Ethics Committee of Psychology (ECP) at the Erasmus University Rotterdam, the Netherlands because the experiment was noninvasive and the results were analyzed anonymously.

## Experiment 1a

### Method

#### Participants

Two hundred participants were recruited via Amazon's Mechanical Turk system of which 189 completed the experiment (102 female, mean age 41, range 18–64). The participants received $1.50 for their participation in the experiment, which lasted approximately 27 min. There were 7 nonnative speakers of English in our sample (Romanian, 2 Dutch, Urdu, Spanish, Marathi, and Hmong). With the exclusion of these participants, our sample included 182 native speakers of English.

#### Stimuli

For the sentence-picture task, a set of 78 sentences and 78 black and white line drawings was used. The 48 experimental sentences were taken from [4]. These sentences described 24 objects, once with an implied horizontal orientation and once with an implied vertical orientation. The 48 experimental pictures, taken from [4] or similar pictures from other sources, represented the same 24 objects as mentioned in the sentences, once in horizontal orientation and once in vertical orientation. Four versions were created, each with 24 sentence-picture pairs, such that orientation matched for half of the pairs and mismatched for the other half and each condition consisted of equal numbers of vertical and horizontal items. Across the four versions, all items were used equally often in the match and mismatch condition. Because all experimental items required a “yes” response, 24 additional sentence-picture pairs were used as fillers. The filler sentences were similar to the experimental sentences in length and position of object nouns, but were followed by an unrelated picture, thus requiring a “no” response. An additional set of 6 sentence-picture pairs (3 related, 3 unrelated) were used as warm-up trials.

An adapted version of the Vividness of Visual Imagery Questionnaire (VVIQ [33]) was used. This questionnaire instructs participants to think of four different situations and then specifies four aspects of the situation that they should try to visualize. Vividness of the mental picture was rated on a scale of 1 = *perfectly clear and vivid as the actual experience* to 5 = *no image at all, you only know that you are thinking of the object* (descriptions for all five intermediate points were also provided). We changed the spelling into American English. In addition, items 9 to 12 were questions about a familiar store (*shop* in the original version), and these were changed so that they corresponded more to contemporary experiences than the original version (*9. The overall appearance of the store from the opposite side of the road or parking lot. 10. The store's name sign including its location, colors, and shape. 11. You are near the entrance. The color, shape, and details of the door. 12. You enter the store and walk into an aisle. You look at the items and pick something you want.*).

#### Procedure

The entire experiment was presented online using the Qualtrics survey software suite. Participants first completed a lexical-decision task with 14 low and 14 high-frequency words. This task was used to familiarize them with the task of making speeded responses to visual stimuli. Next participants completed the sentence-picture task, followed by the imagery questionnaire, followed by a series of questions about participants' notion of the purpose of the experiment, their computer system, the environment in which they took the test as well as demographic questions. The sentence-picture task started with 6 warm-up trials. Following, the 24 experimental and 24 filler trials were presented in a random order. A trial started with a left-justified and vertically centered fixation point for 1000 ms, immediately followed by the sentence, which started at the same location as the fixation point. Participants pressed the p-key when they had understood the sentence. Immediately following the key press a horizontally and vertically centered fixation point appeared for 500 ms, immediately followed by the picture. Participants responded by pressing the a-key for “no” responses or the l-key for “yes” responses. Half of the filler trials were followed by a yes/no comprehension question. The next trial started 500 ms after the response.

The imagery questionnaire was also presented on the computer screen, one item at a time. All response options were presented below the item as buttons with the value of the option (e.g., *Perfectly clear and as vivid as normal vision*), and participants clicked on the button of their choice.

Following the imagery questionnaire participants answered questions about their age, gender, education (on a 7 point scale), native language, the noisiness of their environment (on a 9 point scale) and their computer settings.

### Results

Two participants appeared to have reversed the response keys as they had 0% and 8% correct responses, respectively. Another six participants had accuracy scores <80%, which was three times less than the standard deviation from the mean response accuracy. Data from these participants were also removed. Combined with the removal of the participants who appeared to have reversed the response keys (2), the removal of these 6 participants yielded unequal numbers of participants across lists. To equate the number of participants per list, the last-run participants of three of the lists were removed so that each list had the same number of participants as the shortest list (41). This means that the data analysis involved 164 participants.

The medians are displayed in Table 1. The medians were analyzed with an ANOVA with Match as a within participants factor. There was a small but reliable match advantage of 33 ms, *t*(163) = 2.36, *p* = .02, *BF_01_* = 1.06. Accuracy levels were very high, .98, and did not differ between conditions, *t*<1, *BF_01_* = 15.49.

We also looked at the relation between match effects and imagery ability. For each participant, we calculated the effect sizes of the match effect using the data based on Connell's outlier criteria (because we could not use the medians to calculate individual effect sizes). The effect size was calculated as the difference between the means in the match and mismatch condition, divided by the pooled standard deviations of the match and mismatch conditions. We also calculated each participant's mean score on the VVIQ. Table 2 displays the correlations. The correlation between the VVIQ scores and effect size approached statistical significance, *p* = .06. Effect size did not correlate with age and education level.

**Table 2:** Correlations Between Individual Effects Sizes and Scores on the VVIQ (Vividness of Visual Imagery Questionnaire), Age, and Educational Level.

	VVIQ	Age	Education
Experiment 1a	.15	−.09	.12
Experiment 1b	−.12	−.05	−.01
Experiment 2a	.05	−.09	.04
Experiment 2b	−.06	−.01	−.07
Experiment 3a	.04	−.09	.06
Experiment 3b	−.03	−.07	−.10

Thus, the evidence for a match effect was mixed. The p-values indicated a significant match effect in the RTs, but the Bayes Factor indicated that the RT data provided about as much evidence for the presence and absence of an effect, and the accuracy data even provided about 15 times as much evidence for the null than for the alternative.

## Experiment 1b

### Method

#### Participants

Two-hundred participants were recruited via Amazon's Mechanical Turk system of which 190 completed the experiment (123 female, mean age 31, range 18–63). The participants received $1.00 for their participation in the experiment, which lasted approximately 30 min. There were 6 nonnative speakers of English in our sample (Chinese, Korean, Serbian, Gujarati, Romanian, Hindi). With the exclusion of these participants, our sample included 184 native speakers of English.

#### Stimuli and Procedure

The stimuli and procedure were exactly the same as those in Experiment 1a.

### Results

Three participants had accuracy scores <80%; data from these participants were removed. To equate the number of participants per list, the last-run participants of three of the lists (9) were removed so that each list had the same number of participants as the shortest list (43). This means that the data analysis involved 172 participants.

As shown in Table 1, there was a significant 38 ms match advantage, *t*(171) = 2.61, *p* = .01 *BF_01_* = 0.46. Accuracy was .98 correct in both conditions, |*t*|<1, *BF_01_* = 11.34. Table 2 displays correlations between individual effect sizes and VVIQ, age, and education. Data from one participant were not entered into the correlation analysis because for this participant there were too few observations within Connell's outlier criteria to calculate the SDs and effect size. None of the correlations were significant.

### Discussion

In both Experiments we replicated the published RT result [4]. The match advantages of 33 and 38 ms are comparable to the 44 ms found in the original study. The (small) effect sizes of .10 and .09 are also comparable to that of .13 in the original study. On the other hand, the Bayesian approach indicated that the data provided little evidence for the presence or absence of an effect. Especially with large sample sizes, *p*-values might indicate significance for effects that actually provide just as much evidence for the null-hypothesis as for the alternative [37],[39]. The disadvantage of using *p*-values is that it overestimates evidence against the null with large sample sizes. Fortunately, the Bayes Factor, on the other hand, tends to become more informative with larger sample sizes [40]. Therefore, we combined the data from Experiments 1a and 1b and calculated the Bayes Factor with a larger sample size. For the medians, *BF_01_* = 0.04, indicating substantial evidence for the presence of an effect; that is, the evidence for the alternative hypothesis is about 25 times stronger than that for the null hypothesis. For accuracy, *BF_01_* = 21.95, indicating that there was no effect of match in the accuracy scores, but these were close to ceiling.

The fact that the two MTurk experiments yielded significant effects, however, is meaningful in that the effect was found in noisier conditions than usual, with a much broader participant population (in terms of age and education levels) than in the laboratory. Also of note is that the effect in these experiments cannot be due to experimenter effects, given that the entire experiment was computerized. In sum, the orientation effect is rather small, as also indicated by the Bayes Factor, but with large samples the evidence for an effect was there.

## Experiment 2a

### Method

#### Participants

Two-hundred participants were recruited via Amazon's Mechanical Turk system of which 199 completed the experiment (114 female, mean age 34, range 18–69). The participants received $1.00 for their participation in the experiment, which lasted approximately 28 min. There were 6 nonnative speakers of English in our sample (2 Spanish, Georgian, Japanese, Polish, and Romanian). With the exclusion of these participants, our sample included 193 native speakers of English.

#### Stimuli and Procedure

Experimental stimuli consisted of 56 sentences and 56 pictures, taken from [5]. The sentences described 28 objects, in two different implied shapes. The pictures represented the same 28 objects as mentioned in the sentences, once in the implied shape of one sentence, once in the implied shape of the other sentence. Four versions were created, each with 28 sentence-picture pairs, such that shape matched for half of the pairs and mismatched for the other half. Across the four versions, all items were used equally often in the match and mismatch condition. Because all experimental items required a “yes” response, 28 additional sentence-picture pairs were used as fillers. The filler sentences were similar to the experimental sentences in length and position of object nouns, but were followed by an unrelated picture, thus requiring a “no” response. The procedure was identical to that of Experiment 1a.

### Results

For two participants, the response times were not registered (that is, they were all 0). Two participants appeared to have reversed the response keys as they had between 0% and 4% correct responses. Another 4 participants had accuracy scores <80%, which was three times less than the standard deviation from the mean response accuracy; data from these participants were also removed. Combined with the removal of the participants without RTs (2), the participants who appeared to have reversed the response keys (2), the removal of these 8 participants yielded unequal numbers of participants across lists. To equate the number of participants per list, the last-run participants of three of the lists (9) were removed so that each list had the same number of participants as the shortest list (44). This means that the data analysis involved 176 participants.

As shown in Table 1, there was a significant 57 ms match advantage, *t*(175) = 3.80, *p*<.001, *BF_01_* = 0.02. Accuracy was very high with .98 correct in the match condition and .96 in the mismatch condition; this difference was significant, *t*(175) = 3.43, *p*<.001, *BF_01_* = 0.06. This replicates the published findings [5]. The MTurk participants were considerably slower. This could be due in part to the fact that the MTurk participants were on the average quite a bit older than the lab participants and also did not participate in a controlled environment. Finally, as mentioned earlier, the MTurk participants were slightly more accurate and so may have put more emphasis on accuracy than on speed compared to those who participated in the lab. Nevertheless, despite these differences the original match effect was replicated once more. The Bayes Factor indicates that the evidence for an effect is very strong. Table 2 displays correlations between individual effect sizes and VVIQ, age, and education. None of the correlations were significant.

## Experiment 2b

### Method

#### Participants

Two-hundred participants were recruited via Amazon's Mechanical Turk system of which 188 completed the experiment (117 female, mean age 34, range 18–63). The participants received $1.00 for their participation in the experiment, which lasted approximately 31 min. There were 5 nonnative speakers of English in our sample (Dutch, Spanish, Tamil, Urdu, and Vietnamese). With the exclusion of these participants, our sample included 183 native speakers of English.

#### Stimuli and Procedure

The stimuli and procedure were exactly the same as those in Experiment 2a.

### Results

Three participants had accuracy scores <80%, which was three times less than the standard deviation from the mean response accuracy; data from these participants were removed. To equate the number of participants per list, the last-run participants of three of the lists (4) were removed so that each list had the same number of participants as the shortest list (44). This means that the data analysis involved 176 participants.

As shown in Table 1, there was a significant 70 ms match advantage, *t*(175) = 4.07, *p*<.001, *BF_01_* = 0.01. Accuracy was .98 correct in the match condition and .96 in the mismatch condition, this difference was significant, *t*(175) = 3.94, *p*<.001, *BF_01_* = 0.01. Table 2 displays correlations between individual effect sizes and VVIQ, age, and education. None of the correlations were significant.

The results of Experiments 2a and 2b are very similar and replicate those of previous studies. In all cases participants responded faster and more accurately to pictures when the object matched the shape implied by the sentence then when it mismatched. Moreover, the Bayesian analyses indicated that in both experiments the data provided very strong evidence in favor of a match effect. Analyses of the combined data also resulted in strong evidence, *BF_01_s*<0.01.

## Experiment 3a

In Experiments 3a and 3b pictures matched or mismatched the color implied by the sentence, as Connell [7],[8] investigated.

### Method

#### Participants

Two hundred participants were recruited via Amazon's Mechanical Turk system of which 189 completed the experiment (92 female, mean age 29, range 18–62). The participants received $1.00 for their participation in the experiment, which lasted approximately 25 min. There were 3 nonnative speakers of English in our sample (Tamil, Russian, French). With the exclusion of these participants, our sample included 186 native speakers of English.

#### Stimuli and Procedure

We used the same 24 experimental sentences and 24 pictures as were used by [7],[8]. The sentences consisted of pairs mentioning 12 objects, one sentence implied one color and another implied another color. The pictures depicted the objects in different hues such that each picture matched one sentence and mismatched the other one. Four versions were created, each with 12 sentence-picture pairs, such that color matched for half of the pairs and mismatched for the other half. Across the four versions, all items were used equally often in the match and mismatch condition. Because all experimental items required a “yes” response, 12 additional sentence-picture pairs were used as fillers.

The filler sentences were similar to the experimental sentences in length and position of object nouns, but were followed by an unrelated picture, thus requiring a “no” response. An additional set of 6 sentence-picture pairs (3 related, 3 unrelated) were used as warm-up trials. The procedure was identical to that used in the previous experiments. The filler pictures were similar in style and color to the experimental pictures; they were colored line drawings. The comprehension questions were also new, these were similar to those used by [4].

### Results

Two participants appeared to have reversed the response keys as they had between 0% and 9% correct responses. Given that these participants had no or practically no valid RTs; their data were eliminated. Another 18 participants had accuracy scores <80%; data from these participants were also removed, in accordance with our previous experiments. Combined with the removal of the participants who appeared to have reversed the response keys (2), the removal of these 18 participants yielded unequal numbers of participants across lists. To equate the number of participants per list, the last-run participants of three of the lists (12) were removed so that each list had the same number of participants as the shortest list (38). This means that the data analysis involved 152 participants.

The original studies [7],[8] trimmed the RTs by removing all RTs faster than 300 and slower than 3000 ms and then removed responses that were more than 2 standard deviations from the participant's mean in that condition. This procedure resulted in removal of 6.8% of the correct RTs. There was a 56 ms match advantage [1232 vs. 1288 ms], *t*(151) = 2.31, *p* = .02, *BF_01_* = 1.15. Because we were concerned that the number of observations removed by this procedure is rather high, we conducted an additional analysis, using median RTs as we did for the previous experiments. In this analysis, the match condition was 157 ms faster than the mismatch condition, *t*(151) = 3.91, *p*<.001, *BF_01_* = 0.01. Participants were highly accurate and more so in the match condition than in the mismatch condition, [.96 vs .93]. This 3% difference was significant, *t*(151) = 2.54, *p* = .01, *BF_01_* = 0.68. To compare [7], found .94 and .70 accuracy for match and mismatch respectively, and [8] found .93 accuracy in both conditions. Thus, the match condition was significantly faster and more accurate than the mismatch condition, although the Bayesian analyses indicated that only the median RTs provided evidence for the effect. This result directly contradicts those of the published studies [7],[8], who found significant mismatch advantages (in response times at least). We will discuss this discrepancy further after reporting the replication of this experiment. Table 2 displays correlations between individual effect sizes and VVIQ, age, and education. None of the correlations were significant.

## Experiment 3b

### Method

#### Participants

Two hundred participants were recruited via Amazon's Mechanical Turk system of which 180 completed the experiment (97 female, mean age 31, range 18–69). The participants received $1.00 for their participation in the experiment, which lasted approximately 24 min. There were 3 nonnative speakers of English in our sample (Dutch, Polish, Spanish). With the exclusion of these participants, our sample included 186 native speakers of English.

### Results

Two participants appeared to have reversed the response keys as they had between 9% and 17% correct responses. Another 17 participants had accuracy scores <80%; data from these participants were also removed. Combined with the removal of the participants who appeared to have reversed the response keys, the removal of these participants yielded unequal numbers of participants across lists. To equate the number of participants per list, the last-run participants of three of the lists (6) were removed so that each list had the same number of participants as the shortest list (38). This means that the data analysis involves 152 participants.

Using the outlier-removal plan of Connell, which resulted in removal of 7.9% of the correct RTs, we found no difference between the RTs in the match and mismatch condition [1253 vs. 1256 ms], *t*<1, *BF_01_* = 15.46. As Table 1 shows, however, there was a significant match advantage in the median RTs. The match condition was 85 ms faster than the mismatch condition, *t*(151) = 2.28, *p* = .02, *BF_01_* = 1.22. Accuracy was higher in the match condition, .97 than in the mismatch condition, .92. This difference was significant, *t*(151) = 4.98, *p*<.001, *BF_01_*<0.01. Thus, the match condition was both significantly more accurate and faster than the mismatch condition, although the latter was observed only for the medians and then the Bayesian analyses did not indicate much evidence for an effect. This partly replicates Experiment 3a and again contradicts the published results. Table 2 displays correlations between individual effect sizes and VVIQ, age, and education. None of the correlations were significant.

Comparing Experiments 3a and 3b we see evidence for match advantages, although in Experiment 3a the evidence was mostly in the RTs whereas in Experiment 3b the evidence was mostly in the accuracy. To obtain a better idea of the evidence we combined the data from Experiments 3a and 3b and calculated the Bayes Factor with a larger sample size. Using the trimmed RTs, *BF_01_* = 5.26, which provides substantial evidence for the absence of an effect. For the medians, *BF_01_*<0.01, indicating very strong evidence for the presence of an effect. For accuracy, *BF_01_*<0.01, also indicating very strong evidence for the presence of an effect. The lack of an effect in the trimmed RTs, compared with the analysis of the median RTs shows that the use of Connell's outlier removal procedure eliminated a substantial part of the effect, suggesting that the effect is in the tail of the RT distribution. This contrasts with the match effect in the median RTs, however, because using median RTs also greatly reduces the influence of outlier data. It should also be noted that in this experiment each condition only had six items, which probably resulted in noisier condition means than those in other similar experiments. Another potential issue is that we did not use the original filler items that Connell had used. Using different fillers or comprehension questions might lead to (1) different depths of processing [41], perhaps reliant more on linguistic information rather than simulation aspects of representations, or (2) different strategies adopted by the participants, either of which could lead to attenuation or possibly reversal of effects. However, our filler items were close in all respects to the experimental items, as is common in the sentence-picture verification paradigm. Therefore, it seems unlikely that the use of different fillers is the source of the reversal of Connell's original effect.

## General Discussion

We set out to replicate three well-known findings in the literature on mental simulation, namely on implied orientation [4], shape [5] (Experiment 1), and color [7],[8]. Our replication attempts relied on the population accessible through Amazon's Mechanical Turk. This means that—compared to the typical population of undergraduate psychology students—our participants were much more varied in age and educational background. Therefore, our results have more generalizability across participants and environments than the original experiments. Another advantage of this novel approach is the impossibility of experimenter effects given that the experiments were administered entirely electronically.

Our findings are mixed. For orientation, we replicated the original findings twice. As in the original study [4], the effect was significant but rather small and the Bayesian analyses revealed that only data from a large sample provided substantial evidence for an effect. We speculatively attribute this to the fact that the object's orientation was seldom if ever relevant to the protagonist's actions. We know that comprehenders focus on the causal structure of narratives (causes, effects, goals, outcomes; e.g., [42],[43]). Part of the appeal of the original study [4] was the fact that it appeared to be strongly pitted against finding a match effect. The orientation of the target object is not central to the interpretation of the sentence. Specifically, the orientation has no causal or goal-relevance; it is mostly elaborative in nature. It fleshes out the interpretation of the sentence but does not constrain or afford actions. For example, whether the pencil is horizontally in a drawer or (almost) vertically in a cup does not greatly constrain the actions that can be performed with it. The writing implement is within easy reach and can be used to write, draw, or do anything else with it that one can do with a pencil. Compare this to shape. If an egg is whole it can be broken but not directly eaten. If an egg is sunny side up, it can be eaten, but it cannot be broken. Even if we take a less drastic change in shape, if an eagle is in the sky, its movements have to be tracked differently than when it is in the nest. It is well established in the literature on discourse comprehension that comprehenders use causal and goal information to forge coherent mental representations of the incoming text [42],[44]. Of course the stimuli in the sentence-picture verification paradigm are sentences rather than connected discourse, but it is likely that participants use their natural inclination to make inferences about actions when reading sentences, which would promote representing the (action-relevant) shape of objects but not so much their (action-irrelevant) orientation. We surmise therefore that the relatively small effect size for orientation is due to its limited action relevance (at least in the stimulus sentences). We are currently investigating this idea in a separate set of experiments.

We also provided two successful replications of the original shape effect [5]. The shape effect is stronger than the orientation effect. Unlike the orientation in our stimuli, shape was very often relevant to the action described in a sentence. A live chicken affords different actions than a fried one. Visually tracking a flying eagle is different than observing a perched one. As noted in the Introduction, shape information in general is more diagnostic for categorizing objects than orientation or color. Because of the importance of shape, it is much more likely to be represented in the mental simulation of a sentence. Moreover, a mismatch will have greater impact, because it is more likely to initially suggest a different identity. We considered the possibility that the relative strength of the shape effect is due to the fact that some shape changes are, in fact, category changes. For example, an animal changes into a piece of food. However, only three of the items in our stimulus set were of this type. We also considered whether the reversibility of the object was a factor (a perched eagle can become flying one and vice versa but a broken egg cannot become whole again). We addressed this in an exploratory way by performing item analyses. These analyses suggested that the shape effect is not due to a particular category of items.

Our findings regarding color are puzzling. Not only did we not replicate the original findings by Connell [7],[8], we did, in fact, find the almost perfect opposite pattern. One possibility is that the color effect is weak and will therefore go in any direction, given that the number of color items is small (only 6 items per condition). This is also suggested by the differences in results between median RTs and trimmed RTs. In all experiments that have RTs as dependent measure, reducing the effect of outliers increases statistical power. Different methods are available, and deciding which method is best is rather complicated [45]. We decided to use two different methods because these methods had been used in the original studies. The methods we used differ in how many data points are thrown out (none are thrown out when medians are used), and in this case, with only a few observations per condition, removing a few data points will have a relatively high impact. Another complication is that one of the original studies [7] observed a mismatch advantage in RTs but a huge match advantage in accuracy. Thus, the effect of color match might be too variable to draw any conclusions.

On the other hand, the pattern we observed twice in the experiments reported here is in line with the other findings in the literature and also seems to make more sense theoretically. However, Connell [8] provides some reasons why color might behave differently from orientation and shape; for example, in the color experiments, subjects could also have relied on shape (and to a lesser extent orientation) to make the verification judgments. A reviewer pointed out that if subjects in the color experiments had previously participated in the orientation or shape experiments, they might have come to process objects in a certain way that is different from the Connell experiments. We therefore re-examined the data for the color experiments and excluded subjects who had participated in the orientation or shape experiments.

Fourteen subjects in Experiment 3a had participated in earlier experiments (Experiment 1a through Experiment 2b). Of these, 2 had already been excluded from the data due to other reasons (because of low accuracy and to equate list lengths). This means that 12 of the 152 reported subjects had participated in the shape or orientation experiments. We excluded these participants and where needed supplemented the lists to obtain equal numbers of subjects per list by using subjects who had previously been excluded from the initial analysis to obtain equal numbers per list for that analysis. This left us with 148 subjects. There still was a match advantage (1220 vs. 1380 ms), which was highly reliable (*p* = .0002).

Sixteen subjects in Experiment 3b had participated in earlier experiments. Of these, 4 had already been excluded due to other reasons (low accuracy, end of list). This means that 12 of the 152 subjects had participated in an earlier experiment. We removed the data from these 12 subjects and removed the data from a total of four additional subjects at the end of two of the lists in order to equate the number of subjects per list (it was not possible to supplement the list of subjects with subjects that had previously been removed from the main analysis to create equal numbers per list because the shortest list had no “spare” subjects). This means that the analysis included data from 136 subjects. The match advantage (1197 vs. 1299 ms) remained significant (*p* = .012). These analyses rule out that the effects we obtained were caused by the fact that subjects were focused on shape or orientation due to training in earlier experiments.

An additional important point is that color was action relevant for only a few of the stimuli. For example, the color of a traffic light seems quite relevant but the color of a leaf much less so. As we also argued when discussing the effect of orientation match, mental simulation of color might be much stronger when color is relevant for action than when it is not. Given our own findings as well as theoretical considerations, we conclude that the mental simulation of color in language comprehension deserves further study but that it is likely that future studies will show match advantages rather than mismatch advantages.

An important tenet of mental simulation theory is that mental simulation does not equate mental imagery [2]. Mental imagery is a conscious and resource-consuming process. Mental simulation, on the other hand, is thought to be part-and-parcel of routine cognitive processes. If mental simulation is mental imagery, then one would expect the size of the match effect to correlate with mental imagery ability. We found very little evidence that this is indeed the case.

The work presented here has several methodological implications. The fact that replication attempts apparently sometimes lead to the opposite pattern should give researchers pause and points to the relevance of conducting replications. Mechanical Turk provides a fast and powerful way to conduct replication studies. An added benefit is that it allows one to use much broader samples of participants than are typically available in psychology labs [46]. Our results show that it is even possible to collect meaningful response-time data (see also Appendix S1). Overall, responses are slower than in the lab. This is due in part to the fact that MTurk represent a much larger age range than that of undergraduate students; for example, our samples includes people in their late teens as well as people in their late 60s. In part it is due to the fact that MTurk participants participate in the experiments in environments that may be considerably noisier than the typical lab environment but that are far more representative of natural environments. Finally, all responses were collected through internet connections that may sometimes be slow. Apparently, at least the orientation and shape effects are strong enough to show up under such conditions (and perhaps also the color effect).

We hope to have achieved at least the following three goals. First, we hope to have provided a better assessment of some of the empirical foundations for research on mental simulation in language comprehension. Second, we hope to have made a case for the usefulness of replication attempts in psychological research. Third, we hope to have shown that web-based replications are a fast and efficient tool in this endeavor.

## Supporting Information

Appendix S1 **Validation of data-collection procedure via Mechanical Turk using a lexical-decision task.**
(DOCX)
