# Supplementary material for: Revisiting Mental Simulation in Language Comprehension: Six Replication Attempts
Source: PLoS One. 2012 Dec 26;7(12):e51382. doi: 10.1371/journal.pone.0051382 (PMC3530580; doi:10.1371/journal.pone.0051382)
Supplement: Appendix S1 — Validation of data-collection procedure via Mechanical Turk using a lexical-decision task. (DOCX) [file pone.0051382.s001.docx]

**Appendix**

[46] argued that data collected through MTurk are comparable to those collected in a lab. We used the word frequency effect to ascertain that RTs collected with Qualtrics show similar characteristics as RTs collected directly in the lab. Prior to each of the six experiments reported in this article, participants performed a lexical decision task with 10 high frequency (HF), 10 low-frequency (LF) English words, and 20 pronounceable nonwords. We used this task both as a warm-up task for the sentence-picture verification task and to assess the usefulness of RT data collected with MTurk and Qualtrics. The common finding in the literature is that responses to HF words are reliably faster and more accurate than responses to LF words. This finding was replicated in each of the six experiments. The effect size was large each time and the difference was rather stable across experiments. The difference between HF and LF words varied between 142 ms and 178 ms and the effect was highly significant each time, suggesting that this method of data collection is highly reliable and sensitive.

Table A1.

*Lexical Decision Performance (Median Rts and Accuracy) in Experiments 1a to 3b.**

|  | | HF | | LF | | Difference | p-value | Effect size RT |
| --- | --- | --- | --- | --- | --- | --- | --- | --- |
|  | Accuracy | | RT | Accuracy | RT |  |  |  |
| Exp 1a | .99 | | 750 (233) | .71 | 913 (287) | 163 | <.0001 | .63 |
| Exp 1b | .99 | | 789 (220) | .73 | 953 (425) | 164 | <.0001 | .51 |
| Exp 2a | .99 | | 780 (251) | .72 | 922 (636) | 142 | <.0001 | .46 |
| Exp 2b | .99 | | 838 (558) | .72 | 989 (390) | 151 | <.0001 | .32 |
| Exp 3a | .99 | | 815 (247) | .72 | 992 (331) | 178 | <.0001 | .61 |
| Exp 3b | .99 | | 829 (342) | .72 | 988 (342) | 159 | <.0001 | .47 |

* The results were computed for the same participants whose data were included in the experiments proper. A small number of participants’ (6) lexical decision data could not be used because they produced no correct responses for the low-frequency (LF) words (they misidentified actual words as nonwords).

For comparison, we entered the same words that we had used in our lexical decision task into the English Lexicon Project database (http://elexicon.wustl.edu) and retrieved number of observations, average lexical decision times, and average accuracy scores per word. Lexical decision times were shorter in the ELP database than with our participants (626 ms for HF words and 780 ms for LF words). There can be several reasons for this difference. First, the delivery and time measurement via Qualtrics could have created some slowing for our participants. Second, we did not use a warm-up task for our participants (in fact, the lexical-decision task was a warm-up for the sentence-picture verification task). Third, the fact that our participants did not participate in noise-free lab environments could have played a role. On the other hand, we found no correlation between reported noisiness and response speed in our samples (see below). Fourth, the averages in the ELP are based on relatively small samples (<30 observations per cell) and may thefore not be very stable. Nevertheless, the key finding is that the difference in response times in the ELP sample between HF and LF words of 154 ms is quite comparable to the differences we observed repeatedly, which ranged between 142 and 178 ms. The accuracy scores were .97 for HF words, which is slightly lower than for our participants and .80 for LF words, which is higher.

We also investigated whether age and noisiness of the environment in which participants performed the experiment affected the RTs. We calculated correlations between average RTs per participant, their reported age, and their report of the noisiness of the environment (on a 9-point scale with 1 being no noise at all and 9 being very noisy). For the combined data from Experiments 1a and 1b there was a small, positive correlation between age and RT (.11) but not between noisiness and RT (.05). For Experiments 2a and 2b we found no correlations between RT and age (.05) or noisiness (.00). For Experiments 3a and 3b there was a small, positive correlation between age and RT (.11) but not between noisiness and RT (.00). Thus, this method of data collection appears not to be compromised by the noisiness of the environment and appears sufficiently sensitive to detect differences in response times.
